# Instructional Video and Medical Student Surgical Knot-Tying Proficiency: Randomized Controlled Trial

**DOI:** 10.2196/mededu.9068

**Published:** 2018-04-12

**Authors:** Katarzyna Bochenska, Magdy P Milad, John OL DeLancey, Christina Lewicky-Gaupp

**Affiliations:** ^1^ Division of Female Pelvic Medicine and Reconstructive Surgery Department of Obstetrics and Gynecology Northwestern University Feinberg School of Medicine Chicago, IL United States; ^2^ Division of Minimally Invasive Gynecology Department of Obstetrics and Gynecology Northwestern University Feinberg School of Medicine Chicago, IL United States; ^3^ Division of Female Pelvic Medicine and Reconstructive Surgery Department of Obstetrics and Gynecology University of Michigan Ann Arbor, MI United States

**Keywords:** knot tying, video, proficiency, medical student

## Abstract

**Background:**

Many senior medical students lack simple surgical and procedural skills such as knot tying.

**Objective:**

The aim of this study was to determine whether viewing a Web-based expert knot-tying training video, in addition to the standard third-year medical student curriculum, will result in more proficient surgical knot tying.

**Methods:**

At the start of their obstetrics and gynecology clerkship, 45 students were videotaped tying surgical knots for 2 minutes using a board model. Two blinded female pelvic medicine and reconstructive surgery physicians evaluated proficiency with a standard checklist (score range 0-16) and anchored scoring scale (range 0-20); higher numbers represent better skill. Students were then randomized to either (1) expert video (n=26) or (2) nonvideo (n=24) groups. The video group was provided unlimited access to an expert knot-tying instructional video. At the completion of the clerkship, students were again videotaped and evaluated.

**Results:**

At initial evaluation, preclerkship cumulative scores (range 0-36) on the standard checklist and anchored scale were not significantly different between the nonvideo and video groups (mean 20.3, SD 7.1 vs mean 20.2, SD 9.2, *P*=.90, respectively). Postclerkship scores improved in both the nonvideo and video groups (mean 28.4, SD 5.4, *P*<.001 and mean 28.7, SD 6.5, *P*=.004, respectively). Increased knot board practice was significantly correlated with higher postclerkship scores on the knot-tying task, but only in the video group (*r*=.47, *P*<.05).

**Conclusions:**

The addition of a Web-based expert instructional video to a standard curriculum, coupled with knot board practice, appears to have a positive impact on medical student knot-tying proficiency.

## Introduction

Many senior medical students lack simple surgical and procedural skills such as knot tying [1]. Initiatives including first and second year medical school electives have been proposed to provide early instruction in surgical skills and operating room etiquette [2-4]. The transition from a primarily didactic to a clinically based curriculum between the second and third year of medical school can also be anxiety provoking. In a study performed by Stewart et al [5], medical students entering their clinical years had low levels of confidence and high anxiety related to performing common procedural skills such as knot tying. Following a 4-hour preclinical training course, the students reported increased confidence and proficiency and lowered levels of anxiety. Focused surgical skills electives have also been implemented to help prepare senior medical students for entering residency [6-8].

There is no standardized method of teaching medical students knot-tying skills and several curricula have been proposed [9-11]. Gershuni et al [12] suggested a proficiency-based suturing and knot-tying program early in the fourth year of medical school and Naylor et al [13] demonstrated the benefits of a simulator-based curriculum with third-year medical students. Computer-based video instruction (CBVI) has also been used to teach medical students suturing and knot tying [14-16]. Xeroulis et al [17] demonstrated that medical students taught suturing and knot tying with CBVI showed greater retention of skills at 1 month than controls and students taught by instructors with concurrent or summary feedback. The authors concluded that CBVI could be an efficient and useful adjunct for basic skills training. Similarly, Yeung et al [18] performed a prospective randomized controlled trial comparing the use of text versus video as an education tool for laparoscopic intracorporeal knot tying with medical students. The authors found that the video group achieved superior conceptual understanding of the task compared to the text group.

Additionally, if medical students cannot tie surgical knots, they are often marginalized in the operating room. DiMaggio et al [19] demonstrated the importance of simulation practice in a study evaluating medical students who participated in a 2-day surgical skills laboratory session before starting their surgery clerkship. Students who completed this session expressed that participation in the cadaver laboratory allowed them a greater opportunity to suture in the operating or emergency room during their clerkship.

Overall, in our practice, we have noted that third-year medical students participating in their obstetrics and gynecology clerkship have a dearth of knot-tying experience. Using a prospective, randomized controlled study design, we sought to determine whether having access to an expert knot-tying training video would result in more proficient surgical knot tying.

## Methods

Between November 2015 and March 2016, 55 third-year medical students were approached at the start of their obstetrics and gynecology clerkships for inclusion in this Institutional Review Board-exempt study. As this was an educational intervention, the trial did not require prospective registration.

 As part of the standard curriculum at Northwestern University’s Feinberg School of Medicine in Chicago, IL, all medical students underwent a 1-hour knot-tying education session on the first day of their clerkship. This session involved both didactics and a hands-on knot-tying workshop led by an attending physician. Participating medical students were then randomized to either the standard curriculum (“no video” group) or to the “video” group. Students in the video group received unlimited access to a Web-based expert instructional video on surgical knot tying (courtesy of Dr John OL DeLancey). Students in both groups received access to a knot-tying board for home practice for the duration of their clerkship. At the conclusion of their clerkship, all students received access to the expert knot-tying video.

On the second day of their clerkship, students in both groups were videotaped tying as many square, two-handed knots as they could on a knot-tying board in 2 minutes. Students in both groups also provided demographic (sex, age, race) and prior experience information (number of prior surgical rotations, comfort level with knot tying with range 0-10 and higher numbers indicating more comfort), family members in medicine, and if they were anticipating entering a surgical career. At the conclusion of their 4-week clerkship, students were again videotaped completing the knot-tying task and a satisfaction survey was administered (range 0-10 on nine measures, higher values indicating higher satisfaction with how knot tying was taught during the rotation). Students also self-reported the number of times they had viewed the expert video and practiced knot tying outside of the clinical setting using their knot board.

Videos of students performing the knot-tying tasks were viewed by two blinded female pelvic medicine and reconstructive surgery physicians who evaluated medical student proficiency using a standard knot-tying checklist (score range 0-16) and an anchored scale (range 0-20). The standard knot-tying checklist responses were 1=yes and 2=no on eight knot-tying metrics, including the following: sutures start crossed, index finger lifts suture to form loop, fingers pinch together, push suture through and grasp/tighten, hook thumb under suture, form loop, fingers pinch together, and push suture through and grasp/tighten. The anchored scale was based on a modified objective structured assessment of technical skill scale, which assigned scores from 1 to 5 on four separate procedure domains: respect for tissue, time and motion, instrument handling, and flow of operation and forward planning [20]. Higher scores represented better skills on both metrics. At the completion of the 4-week rotation, all students were again videotaped and evaluated. Statistical analysis was carried out using SPSS version 20 (Chicago, IL, USA). Paired *t* tests, Student *t* tests, Fisher exact, and Pearson correlations were calculated.

## Results

**Figure 1:**
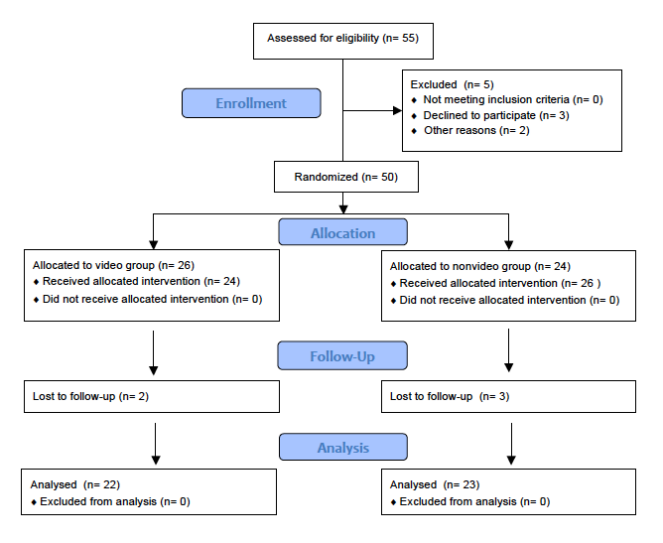
Study enrollment.

Of the initial 55 medical students approached for the study, 3 students declined to participate and 2 transferred from the clerkship. Of the remaining 50 students, 26 students were randomized to the video group and 24 to the nonvideo group. In total, 5 students were lost to follow up and did not complete either of the videotaped tasks. Ultimately, a total of 45 medical students completed both preclerkship and postclerkship knot-tying videotaped tasks and were included in the final analysis: 22 students in the video and 23 students in the nonvideo group (Figure 1).

Participants in the nonvideo and video groups did not differ in age (mean 25.4, SD 1.8 years vs mean 25.0, SD 2.4 years; *P*=.46) or gender (52%, 13/24 female vs 43%, 9/24 female; *P*=.46; Table 1). Students also did not differ in their number of prior surgical rotations (*P*=.52) or median comfort level with knot tying at the start of the rotation (*P*=.55). 

Thirteen of 45 students (29%) in the entire cohort reported having family members who were physicians and 10 students (22%) reported planning on entering surgical fields; this did not differ between groups (*P*=.53 and *P*=.72, respectively). Additionally, preclerkship standard checklist and anchored scale scores on the knot-tying task were not significantly different (*P*=.90) between the two groups.

**Table 1:** Demographics and student-reported measures (N=45).

Variables	Nonvideo group (n=24)	Video group (n=21)	*P* value
Age (years), mean (SD)	25.4 (1.8)	25.0 (2.4)	.46
Sex (female), n (%)	13 (54)	9 (43)	.46
Prior surgical rotations, median (range)	1 (0-4)	1 (0-4)	.52
Comfort level (IQ), median (range)	3 (2-5)	4 (1-5)	.55
Have family members who are physicians, n (%)	8 (33)	5 (24)	.53
Plan to enter surgical field, n (%)	6 (25)	4 (19)	.72

**Table 2:** Knot-tying metrics (N=45).

Scores by group		Prerotation, mean (SD)	Postrotation, mean (SD)	*P* value
**Nonvideo group**				
	Standard checklist	9.7 (3.8)	12.5 (3.1)	.001
	Anchored scale	10.5 (3.7)	15.9 (2.9)	<.001
	Total score	20.3 (7.1)	28.4 (5.4)	<.001
**Video group**				
	Standard checklist	9.0 (4.9)	12.9 (0.7)	.002
	Anchored scale	11.5 (4.8)	15.8 (3.7)	.002
	Total score	20.2 (9.2)	28.7 (6.5)	.001

The median number of self-reported board practice times did not vary significantly between groups (*P*=.66); it was median 5 (range 0-20) in the nonvideo group and median 3 (range 0-20) in the video group. Student performance on the knot-tying tasks as evaluated by the standard checklist and anchored scale improved significantly through the course of the rotation in both groups (Table 2).

For the entire cohort, increased knot board practice was not correlated with higher postclerkship scores on the knot-tying task (*r*=.19, *P*=.23). When stratified by access to the knot-tying video, increased knot board practice was significantly correlated with higher postclerkship scores on the knot-tying task, but only in the video group (*r*=.47, *P*<.05) compared to the nonvideo group (*r*=.11, *P*=.62). Interrater scoring reliability was high (intraclass correlation coefficient 0.98, 95% CI 0.95-0.97). Both the nonvideo and video groups reported high rates of satisfaction with their knot-tying educational experiences (mean 39.0, SD 4.5 vs mean 40.7, SD 3.4, *P*=.17).

## Discussion

In this prospective, randomized controlled study, addition of an expert instructional video to a standard curriculum, coupled with knot board practice, appears to have a positive impact on medical student knot-tying proficiency. These findings suggest that self-directed learning is more effective when augmented with an instructional video. The basic tenants of self-directed medical student learning include diagnosing needs, formulating goals, identifying resources, implementing appropriate activities, and evaluating outcomes [21]. In this study, appropriate activities in learning knot tying included knot board practice outside of the clinical setting, which was augmented with instructional video viewing for half of the study participants.

Self-directed learning is a critical component of modern medical student education. Technical skills, such as knot tying, are increasingly being taught in a simulated environment but additional practice, usually at home, is necessary to achieve task competency. Green et al [8] recently published data suggesting the benefit of home video exercises in teaching technical skills. Additionally, as the costs of operating room time have increased, simulation is becoming an increasingly important adjunct to medical student education [22]. Learning basic skills in a simulation center or practice at home with availability of an educational video, can serve to foster a strong surgical skills’ foundation in medical students. All medical students enrolled in this study were given access to knot-tying boards, which they could use at home. Availability of these resources likely facilitated knot-tying practice outside of the clinical setting.

Our study has several strengths and limitations. We performed a randomized controlled trial to evaluate the role of an expert educational video in medical student knot-tying proficiency. Our study population included medical students who had completed variable amounts of surgical clerkships. Additionally, knot-tying proficiency was evaluated by blinded trained gynecologists in a structured fashion with excellent interrater reliability. Limitations of our study include a relatively small sample size and its focus on a single institution. Because students were asked to record how many times they both viewed the expert video and practiced using the knot board at the conclusion of the rotation, recall bias may be a factor in students’ responses. Future studies may benefit from implementing a logging methodology in which medical students can report their knot board and video use in an ongoing fashion. Additionally, although medical students were advised to not view the video if they were randomized to the nonvideo group, inadvertent crossover may have occurred between the groups.

Based on our analysis, Web-based video instruction appears to be a valuable adjunct to a standard knot-tying medical student curriculum. Additional prospective studies are necessary with focus on addressing the role of knot-tying practice outside of the clinical setting and the availability of practice materials, such as knot-tying boards and instructional videos.

## Abbreviations

CBVI: computer-based video instruction

## Appendix

Multimedia Appendix 1 CONSORT‐EHEALTH checklist (V 1.6.1).
