# Supplementary material for: Instructional Video and Medical Student Surgical Knot-Tying Proficiency: Randomized Controlled Trial
Source: JMIR Med Educ. 2018 Apr 12;4(1):e9. doi: 10.2196/mededu.9068 (PMC5920196; doi:10.2196/mededu.9068)
Supplement: Multimedia Appendix 1 [file mededu_v4i1e9_app1.pdf]

CONSORT-EHEALTH Checklist V1.6.2 Report	Manuscript Number	9068
**(based on CONSORT-EHEALTH V1.6), available at [http://tinyurl.com/consort-ehealth-v1-6].**		
9068		
**Date completed**		
3/18/2018 22:07:23		
**by**		
Bochenska		
The KnOT Study: Knowing Operative Tying		
TITLE		
**1a-i) Identify the mode of delivery in the title**		
Not an issue in this paper		
**1a-ii) Non-web-based components or important co-interventions in title**		
**1a-iii) Primary condition or target group in the title**		
N/A		
ABSTRACT		
**1b-i) Key features/functionalities/components of the intervention and comparator in the METHODS section of the ABSTRACT**		
At the start of their obstetrics and gynecology clerkship, 45 students were videotaped tying surgical knots for 2 minutes using a board model. Two blinded female pelvic medicine and reconstructive surgery physicians evaluated proficiency with a standard checklist (score range 0-16) and anchored scoring scale (range 0-20); higher numbers represent better skill. Students were then randomized to either (1) expert video or (2) no video. The video group was provided unlimited access to an expert knot-tying instructional video. At the completion of the clerkship, students were again videotaped and evaluated.		
**1b-ii) Level of human involvement in the METHODS section of the ABSTRACT**		
At the start of their obstetrics and gynecology clerkship, 45 students were videotaped tying surgical knots for 2 minutes using a board model. Two blinded female pelvic medicine and reconstructive surgery physicians evaluated proficiency with a standard checklist (score range 0-16) and anchored scoring scale (range 0-20); higher numbers represent better skill. Students were then randomized to either (1) expert video or (2) no video. The video group was provided unlimited access to an expert knot-tying instructional video. At the completion of the clerkship, students were again videotaped and evaluated.		
**1b-iii) Open vs. closed, web-based (self-assessment) vs. face-to-face assessments in the METHODS section of the ABSTRACT**		
At the start of their obstetrics and gynecology clerkship, 45 students were videotaped tying surgical knots for 2 minutes using a board model. Two blinded female pelvic medicine and reconstructive surgery physicians evaluated proficiency with a standard checklist (score range 0-16) and anchored scoring scale (range 0-20); higher numbers represent better skill. Students were then randomized to either (1) expert video or (2) no video. The video group was provided unlimited access to an expert knot-tying instructional video. At the completion of the clerkship, students were again videotaped and evaluated.		
**1b-iv) RESULTS section in abstract must contain use data**		
At the start of their obstetrics and gynecology clerkship, 45 students were videotaped tying surgical knots for 2 minutes using a board model. Two blinded female pelvic medicine and reconstructive surgery physicians evaluated proficiency with a standard checklist (score range 0-16) and anchored scoring scale (range 0-20); higher numbers represent better skill. Students were then randomized to either (1) expert video or (2) no video. The video group was provided unlimited access to an expert knot-tying instructional video. At the completion of the clerkship, students were again videotaped and evaluated.		
**1b-v) CONCLUSIONS/DISCUSSION in abstract for negative trials**		
The addition of a Web-based expert instructional video to a standard curriculum, coupled with knot board practice, appears to have a positive impact on medical student knot-tying proficiency.		
INTRODUCTION		
**2a-i) Problem and the type of system/solution**		

Many senior medical students lack simple surgical and procedural skills such as knot tying [1]. Initiatives including first and second year medical school electives have been proposed to provide early instruction in surgical skills and operating room etiquette [2-4]. The transition from a primarily didactic to a clinically based curriculum between the second and third year of medical school can also be anxiety provoking. In a study performed by Stewart et al [5], medical students entering their clinical years had low levels of confidence and high anxiety related to performing common procedural skills such as knot tying. Following a 4-hour preclinical training course, the students reported increased confidence and proficiency and lowered levels of anxiety. Focused surgical skills electives have also been implemented to help prepare senior medical students for entering residency [6-8]. There is no standardized method of teaching medical students knot-tying skills and several curricula have been proposed [9-11]. Gershuni et al [12] suggested a proficiency-based suturing and knot-tying program early in the fourth year of medical school and Naylor et al [13] demonstrated the benefits of a simulator-based curriculum with third-year medical students. Computer-based video instruction (CBVI) has also been used to teach medical students suturing and knot tying [14-16]. Xeroulis et al [17] demonstrated that medical students taught suturing and knot tying with CBVI showed greater retention of skills at 1 month than controls and students taught by instructors with concurrent or summary feedback. The authors concluded that CBVI could be an efficient and useful adjunct for basic skills training. Similarly, Yeung et al [18] performed a prospective randomized controlled trial comparing the use of text versus video as an education tool for laparoscopic intracorporeal knot tying with medical students. The authors found that the video group achieved superior conceptual understanding of the task compared to the text group. Additionally, if medical students cannot tie surgical knots, they are often marginalized in the operating room. DiMaggio et al [19] demonstrated the importance of simulation practice in a study evaluating medical students who participated in a 2-day surgical skills laboratory session before starting their surgery clerkship. Students who completed this session expressed that participation in the cadaver laboratory allowed them a greater opportunity to suture in the operating or emergency room during their clerkship.		
**2a-ii) Scientific background, rationale: What is known about the (type of) system**		
This is not an issue in this manuscript		
**Does your paper address CONSORT subitem 2b?**		
Overall, in our practice, we have noted that third-year medical students participating in their obstetrics and gynecology clerkship have a dearth of knot-tying experience. Using a prospective, randomized controlled study design, we sought to determine whether having access to an expert knot-tying training video would result in more proficient surgical knot tying.		
METHODS		
**3a) CONSORT: Description of trial design (such as parallel, factorial) including allocation ratio**		

Between November 2015 and March 2016, 55 third-year medical students were approached at the start of their obstetrics and gynecology clerkships for inclusion in this Institutional Review Board-exempt study. As part of the standard curriculum at Northwestern University's Feinberg School of Medicine in Chicago, IL, all medical students underwent a 1-hour knot-tying education session on the first day of their clerkship. This session involved both didactics and a hands-on knot-tying workshop led by an attending physician. Participating medical students were then randomized to either the standard curriculum ("no video" group) or to the "video" group. Students in the video group received unlimited access to a Web-based expert instructional video on surgical knot tying (courtesy of Dr John OL DeLancey). Students in both groups received access to a knot-tying board for home practice for the duration of their clerkship. At the conclusion of their clerkship, all students received access to the expert knot-tying video.<br>On the second day of their clerkship, students in both groups were videotaped tying as many square, two-handed knots as they could on a knot-tying board in 2 minutes. Students in both groups also provided demographic (sex, age, race) and prior experience information (number of prior surgical rotations, comfort level with knot tying with range 0-10 and higher numbers indicating more comfort), family members in medicine, and if they were anticipating entering a surgical career. At the conclusion of their 4-week clerkship, students were again videotaped completing the knot-tying task and a satisfaction survey was administered (range 0-10 on nine measures, higher values indicating higher satisfaction with how knot tying was taught during the rotation). Students also self-reported the number of times they had viewed the expert video and practiced knot tying outside of the clinical setting using their knot board.<br>Videos of students performing the knot-tying tasks were viewed by two blinded female pelvic medicine and reconstructive surgery physicians who evaluated medical student proficiency using a standard knot-tying checklist (score range 0-16) and an anchored scale (range 0-20). The standard knot-tying checklist responses were 1=yes and 2=no on eight knot-tying metrics, including the following: sutures start crossed, index finger lifts suture to form loop, fingers pinch together, push suture through and grasp/tighten, hook thumb under suture, form loop, fingers pinch together, and push suture through and grasp/tighten. The anchored scale was based on a modified objective structured assessment of technical skill scale, which assigned scores from 1 to 5 on four separate procedure domains: respect for tissue, time and motion, instrument handling, and flow of operation and forward planning [20]. Higher scores represented better skills on both metrics. At the completion of the 4-week rotation, all students were again videotaped and evaluated. Statistical analysis was carried out using SPSS version 20 (Chicago, IL, USA). Paired t tests, Student t tests, Fisher exact, and Pearson correlations were calculated.		
**3b) CONSORT: Important changes to methods after trial commencement (such as eligibility criteria), with reasons**		
Participants were not included in the trial if they did not complete the clerkship.		

**3b-i) Bug fixes, Downtimes, Content Changes**		
N/A in our study		
**4a) CONSORT: Eligibility criteria for participants**		
Between November 2015 and March 2016, 55 third-year medical students were approached at the start of their obstetrics and gynecology clerkships for inclusion in this Institutional Review Board-exempt study.		
**4a-i) Computer / Internet literacy**		
All medical students were able to access video without difficulty.		
**4a-ii) Open vs. closed, web-based vs. face-to-face assessments:**		
On the second day of their clerkship, students in both groups were videotaped tying as many square, two-handed knots as they could on a knot-tying board in 2 minutes.		
**4a-iii) Information giving during recruitment**		
This study was IRB exempt per our institution		
**4b) CONSORT: Settings and locations where the data were collected**		
Data was collected in an FPMRS office		
**4b-i) Report if outcomes were (self-)assessed through online questionnaires**		
Hand written questionnaires were filled out by the participants		
**4b-ii) Report how institutional affiliations are displayed**		
This was N/A in our study		
**5) CONSORT: Describe the interventions for each group with sufficient details to allow replication, including how and when they were actually administered**		
**5-i) Mention names, credential, affiliations of the developers, sponsors, and owners**		
Not applicable in our study		
**5-ii) Describe the history/development process**		
Not applicable in our study		
**5-iii) Revisions and updating**		
Not applicable in our study		
**5-iv) Quality assurance methods**		
Not applicable in our study		
**5-v) Ensure replicability by publishing the source code, and/or providing screenshots/screen-capture video, and/or providing flowcharts of the algorithms used**		
Not applicable in our study		
**5-vi) Digital preservation**		
Not applicable in our study		
**5-vii) Access**		
Not applicable in our study		
**5-viii) Mode of delivery, features/functionalities/components of the intervention and comparator, and the theoretical framework**		
Not applicable in our study		
**5-ix) Describe use parameters**		
Not applicable in our study		
**5-x) Clarify the level of human involvement**		
Not applicable in our study		
**5-xi) Report any prompts/reminders used**		
Not applicable in our study		
**5-xii) Describe any co-interventions (incl. training/support)**		
Not applicable in our study		

**6a) CONSORT: Completely defined pre-specified primary and secondary outcome measures, including how and when they were assessed**		
Videos of students performing the knot-tying tasks were viewed by two blinded female pelvic medicine and reconstructive surgery physicians who evaluated medical student proficiency using a standard knot-tying checklist (score range 0-16) and an anchored scale (range 0-20). The standard knot-tying checklist responses were 1=yes and 2=no on eight knot-tying metrics, including the following: sutures start crossed, index finger lifts suture to form loop, fingers pinch together, push suture through and grasp/tighten, hook thumb under suture, form loop, fingers pinch together, and push suture through and grasp/tighten. The anchored scale was based on a modified objective structured assessment of technical skill scale, which assigned scores from 1 to 5 on four separate procedure domains: respect for tissue, time and motion, instrument handling, and flow of operation and forward planning [20]. Higher scores represented better skills on both metrics. At the completion of the 4-week rotation, all students were again videotaped and evaluated. Statistical analysis was carried out using SPSS version 20 (Chicago, IL, USA). Paired t tests, Student t tests, Fisher exact, and Pearson correlations were calculated.		
**6a-i) Online questionnaires: describe if they were validated for online use and apply CHERRIES items to describe how the questionnaires were designed/deployed**		
Not applicable in our study		
**6a-ii) Describe whether and how “use” (including intensity of use/dosage) was defined/measured/monitored**		
Not applicable in our study		
**6a-iii) Describe whether, how, and when qualitative feedback from participants was obtained**		
Not applicable in our study		
**6b) CONSORT: Any changes to trial outcomes after the trial commenced, with reasons**		
Data was collected in an FPMRS office		
**7a) CONSORT: How sample size was determined**		
**7a-i) Describe whether and how expected attrition was taken into account when calculating the sample size**		
Not applicable in our study		
**7b) CONSORT: When applicable, explanation of any interim analyses and stopping guidelines**		

Videos of students performing the knot-tying tasks were viewed by two blinded female pelvic medicine and reconstructive surgery physicians who evaluated medical student proficiency using a standard knot-tying checklist (score range 0-16) and an anchored scale (range 0-20). The standard knot-tying checklist responses were 1=yes and 2=no on eight knot-tying metrics, including the following: sutures start crossed, index finger lifts suture to form loop, fingers pinch together, push suture through and grasp/tighten, hook thumb under suture, form loop, fingers pinch together, and push suture through and grasp/tighten. The anchored scale was based on a modified objective structured assessment of technical skill scale, which assigned scores from 1 to 5 on four separate procedure domains: respect for tissue, time and motion, instrument handling, and flow of operation and forward planning [20]. Higher scores represented better skills on both metrics. At the completion of the 4-week rotation, all students were again videotaped and evaluated. Statistical analysis was carried out using SPSS version 20 (Chicago, IL, USA). Paired t tests, Student t tests, Fisher exact, and Pearson correlations were calculated.		
**8a) CONSORT: Method used to generate the random allocation sequence**		
Participating medical students were then randomized to either the standard curriculum (“no video” group) or to the “video” group		
**8b) CONSORT: Type of randomisation; details of any restriction (such as blocking and block size)**		
Block randomization		
**9) CONSORT: Mechanism used to implement the random allocation sequence (such as sequentially numbered containers), describing any steps taken to conceal the sequence until interventions were assigned**		
Division randomly into 2 groups by random division of group		
**10) CONSORT: Who generated the random allocation sequence, who enrolled participants, and who assigned participants to interventions**		
Study coordinator		
**11a) CONSORT: Blinding - If done, who was blinded after assignment to interventions (for example, participants, care providers, those assessing outcomes) and how**		
**11a-i) Specify who was blinded, and who wasn’t**		
All evaluators were blinded		
**11a-ii) Discuss e.g., whether participants knew which intervention was the “intervention of interest” and which one was the “comparator”**		
Yes participants were aware		
**11b) CONSORT: If relevant, description of the similarity of interventions**		
Not applicable in this study		
**12a) CONSORT: Statistical methods used to compare groups for primary and secondary outcomes**		
Paired t tests, Student t tests, Fisher exact, and Pearson correlations were calculated.		
**12a-i) Imputation techniques to deal with attrition / missing values**		
Was not an issues in this study		
**12b) CONSORT: Methods for additional analyses, such as subgroup analyses and adjusted analyses**		
Was not an issue in this study		
RESULTS		
**13a) CONSORT: For each group, the numbers of participants who were randomly assigned, received intended treatment, and were analysed for the primary outcome**		

Of the initial 55 medical students approached for the study, three did not complete the clerkship and seven did not complete either of the videotaped tasks. Ultimately, a total of 45 medical students completed both preclerkship and postclerkship knot-tying videotaped tasks and were included in the final analysis. Participants in the nonvideo and video groups did not differ in age (mean 25.4, SD 1.8 years vs mean 25.0, SD 2.4 years; P=.46) or gender (52%, 13/24 female vs 43%, 9/24 female; P=.46) (Table 1). Students also did not differ in their number of prior surgical rotations (P=.52) or median comfort level with knot tying at the start of the rotation (P=.55). Thirteen of 45 students (29%) in the entire cohort reported having family members who were physicians and 10 students (22%) reported planning on entering surgical fields; this did not differ between groups (P=.53 and P=.72, respectively). Additionally, preclerkship standard checklist and anchored scale scores on the knot-tying task were not significantly different (P=.90) between the two groups.		
**13b) CONSORT: For each group, losses and exclusions after randomisation, together with reasons**		
Of the initial 55 medical students approached for the study, three did not complete the clerkship and seven did not complete either of the videotaped tasks. Ultimately, a total of 45 medical students completed both preclerkship and postclerkship knot-tying videotaped tasks and were included in the final analysis. Participants in the nonvideo and video groups did not differ in age (mean 25.4, SD 1.8 years vs mean 25.0, SD 2.4 years; P=.46) or gender (52%, 13/24 female vs 43%, 9/24 female; P=.46) (Table 1). Students also did not differ in their number of prior surgical rotations (P=.52) or median comfort level with knot tying at the start of the rotation (P=.55). Thirteen of 45 students (29%) in the entire cohort reported having family members who were physicians and 10 students (22%) reported planning on entering surgical fields; this did not differ between groups (P=.53 and P=.72, respectively). Additionally, preclerkship standard checklist and anchored scale scores on the knot-tying task were not significantly different (P=.90) between the two groups.		
**13b-i) Attrition diagram**		
Not an issue in this study		
**14a) CONSORT: Dates defining the periods of recruitment and follow-up**		
Between November 2015 and March 2016, 55 third-year medical students were approached at the start of their obstetrics and gynecology clerkships for inclusion in this Institutional Review Board-exempt study		
**14a-i) Indicate if critical “secular events” fell into the study period**		
Not an issue in this study		
**14b) CONSORT: Why the trial ended or was stopped (early)**		
Not an issue in this study		
**15) CONSORT: A table showing baseline demographic and clinical characteristics for each group**		
Yes Table 1		
**15-i) Report demographics associated with digital divide issues**		

Not an issue in this paper		
**16a) CONSORT: For each group, number of participants (denominator) included in each analysis and whether the analysis was by original assigned groups**		
**16-i) Report multiple "denominators" and provide definitions**		
Of the initial 55 medical students approached for the study, three did not complete the clerkship and seven did not complete either of the videotaped tasks. Ultimately, a total of 45 medical students completed both preclerkship and postclerkship knot-tying videotaped tasks and were included in the final analysis. Participants in the nonvideo and video groups did not differ in age (mean 25.4, SD 1.8 years vs mean 25.0, SD 2.4 years; P=.46) or gender (52%, 13/24 female vs 43%, 9/24 female; P=.46) (Table 1). Students also did not differ in their number of prior surgical rotations (P=.52) or median comfort level with knot tying at the start of the rotation (P=.55). Thirteen of 45 students (29%) in the entire cohort reported having family members who were physicians and 10 students (22%) reported planning on entering surgical fields; this did not differ between groups (P=.53 and P=.72, respectively). Additionally, preclerkship standard checklist and anchored scale scores on the knot-tying task were not significantly different (P=.90) between the two groups.		
**16-ii) Primary analysis should be intent-to-treat**		
Not an issue in this paper		
**17a) CONSORT: For each primary and secondary outcome, results for each group, and the estimated effect size and its precision (such as 95% confidence interval)**		
Not an issue in this paper		
**17a-i) Presentation of process outcomes such as metrics of use and intensity of use**		
Not an issue in this paper		
**17b) CONSORT: For binary outcomes, presentation of both absolute and relative effect sizes is recommended**		
Not an issue in this paper		
**18) CONSORT: Results of any other analyses performed, including subgroup analyses and adjusted analyses, distinguishing pre-specified from exploratory**		
Not an issue in this paper		
**18-i) Subgroup analysis of comparing only users**		
Not an issue in this paper		
**19) CONSORT: All important harms or unintended effects in each group**		
Not an issue in this paper		
**19-i) Include privacy breaches, technical problems**		
Not an issue in this paper		
**19-ii) Include qualitative feedback from participants or observations from staff/researchers**		
Not an issue in this paper		
DISCUSSION		
**20) CONSORT: Trial limitations, addressing sources of potential bias, imprecision, multiplicity of analyses**		
**20-i) Typical limitations in ehealth trials**		

Our study has several strengths and limitations. We performed a randomized controlled trial to evaluate the role of an expert educational video in medical student knot-tying proficiency. Our study population included medical students who had completed variable amounts of surgical clerkships. Additionally, knot-tying proficiency was evaluated by blinded trained gynecologists in a structured fashion with excellent interrater reliability. Limitations of our study include a relatively small sample size and its focus on a single institution. Because students were asked to record how many times they both viewed the expert video and practiced using the knot board at the conclusion of the rotation, recall bias may be a factor in students' responses. Future studies may benefit from implementing a logging methodology in which medical students can report their knot board and video use in an ongoing fashion. Additionally although medical students were advised to not view the video if they were randomized to the nonvideo group, inadvertent crossover may have occurred between the groups.		
**21) CONSORT: Generalisability (external validity, applicability) of the trial findings**		
**21-i) Generalizability to other populations**		
Based on our analysis, Web-based video instruction appears to be a valuable adjunct to a standard knot-tying medical student curriculum.		
**21-ii) Discuss if there were elements in the RCT that would be different in a routine application setting**		
Based on our analysis, Web-based video instruction appears to be a valuable adjunct to a standard knot-tying medical student curriculum.		
**22) CONSORT: Interpretation consistent with results, balancing benefits and harms, and considering other relevant evidence**		
**22-i) Restate study questions and summarize the answers suggested by the data, starting with primary outcomes and process outcomes (use)**		
In this prospective, randomized controlled study, addition of an expert instructional video to a standard curriculum, coupled with knot board practice, appears to have a positive impact on medical student knot-tying proficiency. These findings suggest that self-directed learning is more effective when augmented with an instructional video.		
**22-ii) Highlight unanswered new questions, suggest future research**		
Additional prospective studies are necessary with focus on addressing the role of knot-tying practice outside of the clinical setting and the availability of practice materials, such as knot-tying boards and instructional videos.		
Other information		
**23) CONSORT: Registration number and name of trial registry**		
IRB exempt study at Northwestern University		
**24) CONSORT: Where the full trial protocol can be accessed, if available**		
Can be accessed by request from the author		
**25) CONSORT: Sources of funding and other support (such as supply of drugs), role of funders**		
No funding		
**X26-i) Comment on ethics committee approval**		
Between November 2015 and March 2016, 55 third-year medical students were approached at the start of their obstetrics and gynecology clerkships for inclusion in this Institutional Review Board-exempt study		
**x26-ii) Outline informed consent procedures**		

Between November 2015 and March 2016, 55 third-year medical students were approached at the start of their obstetrics and gynecology clerkships for inclusion in this Institutional Review Board-exempt study		
**X26-iii) Safety and security procedures**		
Between November 2015 and March 2016, 55 third-year medical students were approached at the start of their obstetrics and gynecology clerkships for inclusion in this Institutional Review Board-exempt study		
**X27-i) State the relation of the study team towards the system being evaluated**		
No conflicts of interest		
